# Mucosal Application of gp140 Encoding DNA Polyplexes to Different Tissues Results in Altered Immunological Outcomes in Mice

**DOI:** 10.1371/journal.pone.0067412

**Published:** 2013-06-24

**Authors:** Jamie F. S. Mann, Paul F. McKay, Samantha Arokiasamy, Reeyeshkumar K. Patel, John S. Tregoning, Robin J. Shattock

**Affiliations:** 1 Section of Infectious Diseases, Department of Medicine, Imperial College London, London, United Kingdom; 2 Centre for Infection, Division of Clinical Sciences, St Georges University of London, London, United Kingdom; Deakin University, Australia

## Abstract

Increasing evidence suggests that mucosally targeted vaccines will enhance local humoral and cellular responses whilst still eliciting systemic immunity. We therefore investigated the capacity of nasal, sublingual or vaginal delivery of DNA-PEI polyplexes to prime immune responses prior to mucosal protein boost vaccination. Using a plasmid expressing the model antigen HIV CN54gp140 we show that each of these mucosal surfaces were permissive for DNA priming and production of antigen-specific antibody responses. The elicitation of systemic immune responses using nasally delivered polyplexed DNA followed by recombinant protein boost vaccination was equivalent to a systemic prime-boost regimen, but the mucosally applied modality had the advantage in that significant levels of antigen-specific IgA were detected in vaginal mucosal secretions. Moreover, mucosal vaccination elicited both local and systemic antigen-specific IgG^+^ and IgA^+^ antibody secreting cells. Finally, using an Influenza challenge model we found that a nasal or sublingual, but not vaginal, DNA prime/protein boost regimen protected against infectious challenge. These data demonstrate that mucosally applied plasmid DNA complexed to PEI followed by a mucosal protein boost generates sufficient antigen-specific humoral antibody production to protect from mucosal viral challenge.

## Introduction

Mucosal surfaces act as the first line of defence against a plethora of different opportunistic pathogens including infectious agents of the respiratory, gastrointestinal and the genitourinary tracts [1]. Apart from a few licensed mucosally applied vaccines, the vast majority of current vaccination strategies employ systemic routes of immunisation, thought to be less effective in generating protective local responses at mucosal surfaces [1]. In contrast, mucosal vaccination has been shown to effect local and systemic immune responses. This is because the site of antigen entry can play a part in the T and B cell receptor imprinting and thus their homing capabilities [2], [3]. Furthermore, the delivery site of mucosally-applied vaccine formulations has been shown to impact immune outcome [4]. Despite this, a major impediment to the development of vaccines targeting mucosal surfaces is that the direct application of antigens to mucosal surfaces results in weak immune responses [5]. Hence newer vaccine delivery technologies, capable of utilising or circumventing the formidable mucosal barrier and initiating the desired immune responses, have the potential to drive the field of mucosal vaccination forward.

Currently, most clinically approved vaccines rely on the production of protective humoral responses. However genetic based vaccines have been shown to induce both the cellular and humoral arms of immunity [6]. To do this, DNA vaccines utilise the recipients host cell machinery to manufacture the encoded transgene product for major histocompatibility complex (MHC) class I and II presentation [7]. This process results in the generation of endogenous vaccinating proteins that are conformationally similar to the natively expressed form of the antigen and with the appropriate post-translational modifications [8], [9]. Despite this, the delivery of vaccinating DNA *in vivo* has resulted in limited transgene expression [10], normally in the nanogram range [11] leading to reduced immunogenicity in larger animal models or human clinical trials [8]. To circumvent these short fallings, DNA vaccinations have been incorporated into prime-boost vaccination regimens. Critically, the use of DNA prime vaccinations in a number of prime-boost studies has been shown to broaden both the pathogen-specific humoral and cellular immune responses, an outcome that is likely to enhance the efficacy of any prophylactic vaccine [8], [12], [13].

Within this study we set out to improve upon current prophylactic mucosal vaccine regimens by applying a vaccine prime topically to the mucosa using a DNA preparation incorporating polyethyleneimine (PEI). Condensation of plasmid DNA with cationic PEI has previously shown great potential in the vaccine delivery field by significantly increasing transfection rates and immune responses [14]–[16]. Here we sought to investigate the potential of a DNA prime – protein boost vaccination strategy to elicit humoral antibody responses when applied to three different mucosal surfaces. Specifically we compared the immunogenicity of nasal, vaginal and sublingual routes of DNA (HIV-1 gp140) polyplex prime vaccination followed by protein boost vaccination with recombinant HIV-1 gp140. We show that plasmid DNA followed with recombinant protein, delivered via the nasal and sublingual routes, elicited strong serum and mucosal antigen-specific antibody responses and significant numbers of antigen-specific IgG^+^ and IgA^+^ antibody secreting cells in the spleen. However, vaginal vaccination elicited serum and mucosal antigen-specific IgA in the absence of detectable specific IgG while also increasing numbers of locally resident specific IgA^+^ B cells. In addition, we show that the IgG antibody bias is influenced by the route of DNA mucosal priming where sublingual immunisation displayed a higher tendency or bias toward an IgG1 response while nasal immunisation generated a more balanced response. Finally, we demonstrate that intranasally applied DNA prime immunisation and recombinant protein boost vaccination is sufficient to protect mice from influenza infection.

## Materials and Methods

### Protein, DNA Plasmid and Complex Formation

The HIV-1 CN54-gp140 clade C/B (codon optimised) was provided by Roger Tatoud, UK HVC, Imperial College London. The ZM96-gp140 clade C plasmid was provided by Simon Jeffs, Imperial College London. The Influenza A virus (A/Aichi/2/1968(H3N2)) hemagglutinin (X31-HA) gene (GenBank accession no. CY121117.1) was synthesised and codon optimised for maximal expression in mice using the OptimumGene™ algorithm (GenScript, CN). The X31-HA gene sequence was altered in accordance to the work published by Wei *et al*., 2009 [17], where the HA gene was modified at the C terminus through the addition of the bacteriophage T4 fibritin foldon trimerization sequence. The X31-HA insert was cloned into the pmaxFP™-Red C vector (Lonza, UK). Large scale plasmid production was carried out using an Endo free Gigaprep kit (Qiagen, UK). The plasmids were then complexed to InVivoJet PEI (PolyPlus Transfection, Fr) using an N/P ratio of 8 as per the manufacturer’s instructions. Homologous recombinant CN54-gp140 protein was purchased from Polymun (Austria) and the H3N2 A/Aichi/2/1968 was purchased from Sino Biological Inc., (China).

### Immunisation, Sampling and Infections

Female BALB/c mice (Harlan, UK), 6–8 weeks old, were placed into groups of n = 8 or n = 6 and housed in a fully acclimatised room. All animals were handled and procedures performed in accordance with the terms of a project licence granted under the UK Home Office Animals (Scientific Procedures) Act 1986. One week prior to the commencement of the study and every three weeks thereafter, mice receiving vaginal immunisations were treated subcutaneously with 2 mg medroxy-progesterone (Pharmacia, UK) to synchronise the menstrual cycle and thin the vaginal epithelium. Food and water were supplied *ad libitum*. For initial studies, mice received DNA with (+PEI) or without (-PEI) to determine the utility of cation complex. Subsequently, all mice received three 20 µg/15 µl doses of CN54-gp140 DNA-PEI formulation vaginally (I.Vag), nasally (IN) or sublingually (SL) followed by three 20 µg CN54-gp140 protein boosts by the same route. Intramuscular (IM) positive controls received three 20 µg/50 µl doses of naked plasmid DNA into the right quadriceps followed by three 20 µg/50 µl gp140 protein boosts in the same muscle. For HA vaccine studies, 20 µg of HA DNA polyplex or protein was delivered in a 30 µl volume and administered via the intranasal, sublingual and vaginal routes.

Tail bleeds were collected weekly without anti-coagulant and centrifuged in a Heraeus Biofuge pico (Fisher, UK) at 1000 g for 20 min. The serum was harvested and transferred into fresh 0.5 ml micro-centrifuge tubes (Starlab, UK), and stored at −20°C until antibody titres were determined by ELISA. Vaginal lavage using three 25 µl washes/mouse with PBS, that were then pooled, was carried out weekly. Lavage samples were incubated for 30 min with 4 µl protease inhibitor (Roche Diagnostics, Germany) before centrifuging at 1000 g for 10 min. The fluid supernatant from these treated samples were then transferred into fresh 0.5 ml micro-centrifuge tubes, and stored at −20°C until antibody titres were determined by ELISA. For infections mice were lightly anesthetised using isoflurane before nasal instillation of 100 µl of 5HAU Influenza X-31 (H3N2). Mice weights were monitored daily for signs of infection.

### Antibody ELISA

An in-house developed, quantitative, CN54gp140 and HA antigen-specific antibody ELISA protocol was followed. Briefly, ELISA plates were coated with 100 µl per well of 5 µg/ml gp140 antigen or purified recombinant HA diluted in sterile PBS. Plates were then covered with a plate seal and incubated overnight at 4°C. The plates were then washed four times by PBST (Invitrogen, UK) and blocked by adding 200 ul per well of assay buffer (1% BSA in 0.05% PBST). The plates were then sealed and incubated for 1 hour at 37°C. After the incubation, the plates are washed 4 times with 250 µl of PBST. A 50 µl volume of antibody sample from serum diluted 1∶1000, 1∶10,000 and 1∶50,000 in sample diluent were then added to the plate in triplicate. Plates were than resealed and incubated for 1 hour at 37°C. The plates were washed 4 times with 250 µl of PBST per well, before the addition of a 1∶4000 dilution in sample diluent of 100 µl/well of either anti-mouse IgG-HRP, anti-mouse IgG1-HRP, anti-mouse IgG2a-HRP or IgA-HRP (Southern Biotech) to detect antigen reactive antibody. Plates were incubated for 1 hour at 37°C before 4 washes with 250 µl PBST. Plates were developed using the addition of 100 µl/well TMB and the reaction stopped after 5 min using Stop solution (Insight Biotechnologies, UK). Standards on the plate consisted of coating with 100 µl anti-mouse Kappa (1∶3200) and Lambda (1∶3200) light chain (Serotec, UK), blocking as before and then adding 50 µl of highly purified polyclonal mouse IgG, IgG1, IgG2a or IgA (Southern Biotech, UK) in a 1∶3 dilution in sample diluent down the plate starting at 200 ng/ml. The rest of the process was the same as for the samples where the secondary antibodies were incubated for 1 hour, washed, the reaction developed with TMB and stopped with Stop solution (Insight Biotechnologies, UK). Mucosal samples were assayed in duplicates using a 1∶10, 1∶50 and 1∶250 dilution. For mucosal IgA, biotinylated-IgA (1∶4000) was used followed by one hour incubation with Streptavidin-HRP (R&D systems). The absorbance was read on a KC4 Spectrophotometer at 450 nm (Bio-Tek Industries, Inc).

### Tissue Processing for ELISPOT Assay

To assess IgG, IgA B cell and IFN-γ T cell responses, lymphocyte cultures from spleens or vaginas of immunised and control mice were made. Briefly, mice were euthanised, and their spleens removed aseptically and placed into individual 20 ml universal tubes (Greiner, UK) containing 2 ml RPMI 1640 medium (Sigma Aldrich Ltd, UK). The vaginas were then removed and pooled together according to their groups. The spleens were placed into Petri dishes and single cell suspensions made by disrupting the spleens. This was achieved by grinding the spleens through a 70 µm Nylon Cell Strainers (BD Falcon, UK) using a syringe plunger. The cell suspensions were then centrifuged at 350 g for 10 min. The supernatants were decanted and the pelleted cells re-suspended in ACK lysis buffer for 5 min (Gibco, UK). The cell suspensions were vortexed and centrifuged at 350 g for 10 min. The pelleted cells were decanted and re-suspended in 1 ml RPMI 1640 medium. This step was repeated three times with the cells resuspended in complete RPMI and then filtered through a 100 µm Filcon unit (BD Biosciences, UK). The cells were counted using a hemocytometer. For the pooled vaginas the RPMI media was decanted and the vaginas cut into small pieces (2×2 mm) and mechanically disrupted using scalpels. The pooled samples were then incubated with enzymatic digestion solution containing Liberase DL (15 ug/ml, Roche) and DNase (100 U/ml, Roche) in RPMI, for 45 min at 37°C. The digested tissue was then placed in sterile 50 µm Medicon grinders before being homogenised in a Medimachine (BD Biosciences, UK) for 2 min. Samples were visually inspected every 30 s to prevent intact material remaining on the blades. The homogenate was extracted and the samples filtered through a 100 µm Filcon unit before cell counting.

### Antigen-specific ELISPOT

Antigen-specific IgG, and IgA spleen and vaginal ELISPOT assays were carried out using a commercially available ELISPOT kit with associated detection antibodies and reagents (MABTECH, UK) as per the manufacturer’s instructions. Briefly, plates were activated using two washes of 50 µl/well of 70% ethanol followed by five rinses with 200 µl/well sterile water. The plates were then coated using 5 µg/ml gp140 antigen in PBS overnight at 4°C. The next day, plates were washed five times in sterile PBS and blocked for 30 minutes using complete RPMI. The media was removed from the plate, the cells at a concentration of 5×10^6^ cells/ml added, and the plates incubated for 16 hours at 37°C supplemented with 5% CO_2_. The next day, plates were washed five times in PBS before a two hour incubation with 100 µl/well of 1 µg/ml of the supplied biotinylated anti-mouse IgG. The plates were washed five times before adding a 1/1000 dilution of the supplied Streptavidin-ALP for one hour. Samples were developed using the kits BCIP/NBT-plus reagent. For antigen-specific IgA ELISPOT samples, a biotinylated anti-IgA was used. For assessment of IFN-γ T cell responses, a similar protocol was followed except 15 µg/ml coating antibody (AN18) was used to coat the plates while 5 µg/ml of PHA polyclonal activator served as the positive controls for each sample. Cell cultured in complete RPMI in the absence of stimuli served to demonstrate background levels of activation. To detect spots, biotinylated anti-IFN-γ antibody was added at 1 µg/ml for 2 hours before washing and incubating with Streptavidin-HRP for 1 hour. The plate was washed as before and 100 µl/well TMB substrate added. ELISPOT plates were read using an AID ELISPOT reader ELR03 (Autoimmun Diagnostika GmbH, Ger) and the AID ELISPOT READER 4 software (Autoimmun Diagnostika GmbH, Ger). The background of the IgG, IgA and IFN-γ ELISPOT assays were calculated as twice the mean number of SFU/million cells in the un-stimulated samples. After subtracting the background, samples yielding 50 SFU/million or more were regarded as positive.

### Statistical Analysis

Statistical analysis of the data was carried out using a Mann–Whitney, *U*-test or Wilcoxon non-parametric paired T test using GraphPad PRISM software.

## Results

### A DNA Priming Strategy Delivered via the Nasal Route is Superior to Intramuscular Vaccination for the Generation of Mucosal IgA

**Figure 1:**
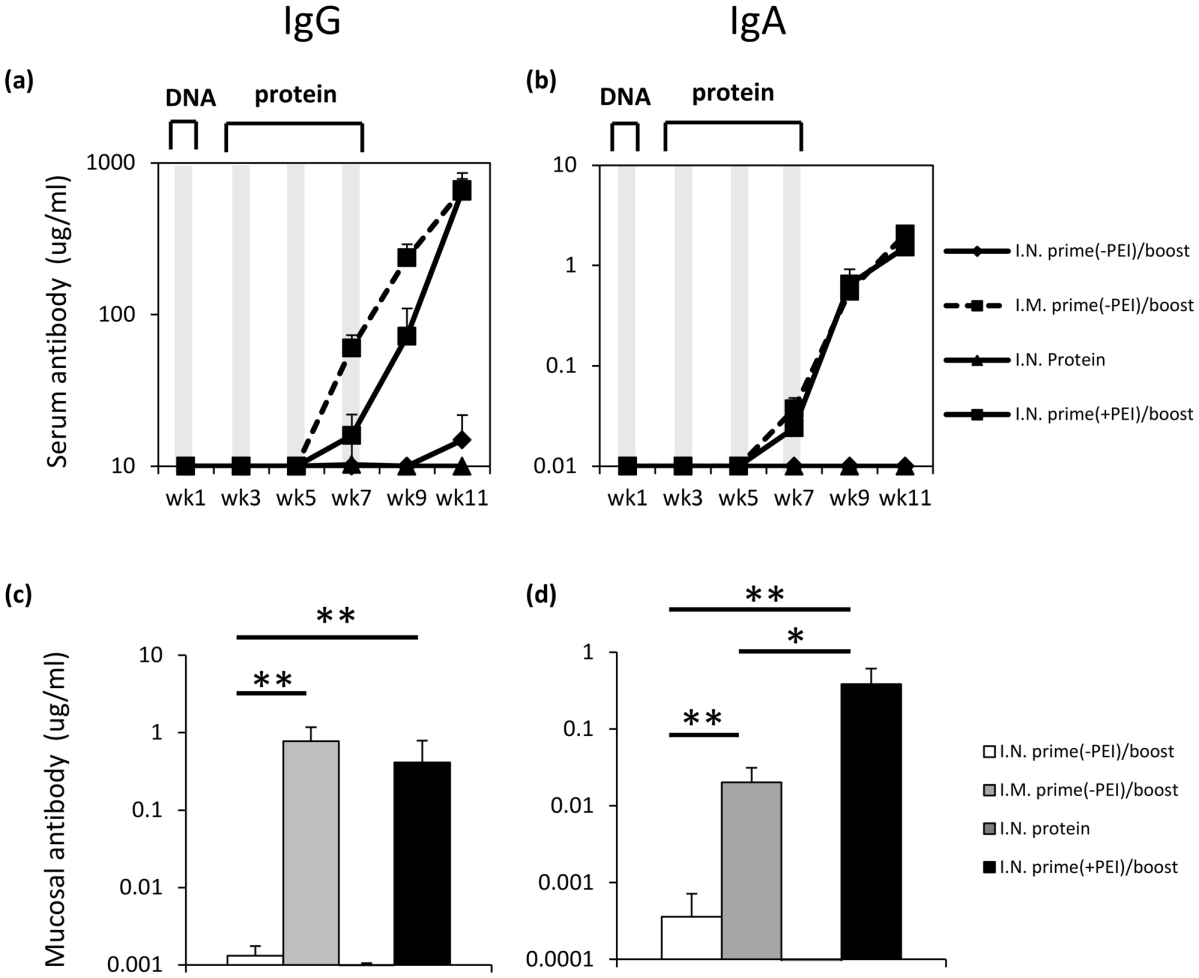
A single nasal DNA prime, triple protein boost regimen results in antigen-specific IgG and IgA in serum samples. Mice (n = 5 per group) were vaccinated with either a single 20 µg DNA IN prime vaccination using ZM96-gp140 (+ PEI) followed by three 20 µg gp140 protein boost vaccinations (▪), three 20 µg gp140 protein boost vaccinations (▴), a single 20 µg DNA IM prime vaccination using ZM96-gp140 (− PEI) followed by three 20 µg gp140 protein boost vaccinations (-▪-) and finally a single 20 µg DNA IN prime vaccination using ZM96-gp140 (− PEI) followed by three 20 µg gp140 protein boost vaccinations (♦). Immunisations are indicated by vertical shaded lines. Antigen-specific serum IgG (**a**) and IgA (**b**) were assessed by ELISA one week after each immunisation until study week 11. Vaginal mucosal lavage was performed to evaluate antigen-specific IgG (**c**) and IgA (**d**) antibody responses during study week 11. Antibody results are expressed as geometric group means (µg/ml+SEM). Statistical significance was assessed using Mann Whitney U test with **p<0.005; *p<0.05. Data are representative of two independent experiments.

Initial experiments were performed to assess the ability of a trimeric gp140 DNA polyplex vaccine formulation to prime mucosal immune responses after topical nasal (IN) application. This was undertaken to determine whether mucosal priming with a gp140 expressing plasmid could be boosted by CN54-gp140 protein applied in the absence of adjuvant by the same route (**Fig. 1**). A single DNA prime of 20 µg of gp140 expressing DNA was administered either alone (-PEI) or complexed to PEI (+PEI), followed by three fortnightly CN54-gp140 protein boost vaccinations (**Fig. S1**). After the initial DNA prime and protein boost immunisations, no detectable antigen-specific IgG (**Fig. 1a**) or IgA (**Fig. 1b**) was recorded in serum samples. However, after a second protein boost vaccination, elevated antibody levels were detected for both the IN prime(+PEI)/boost and IM prime(-PEI)/boost groups. A third and final protein boost vaccination generated a peak antigen-specific IgG concentration of 664 ug/ml [IN prime(+PEI)/boost] and 648 ug/ml [IM prime(-PEI)/boost] and a peak specific IgA concentration of 1.51 ug/ml [IN prime(+PEI)/boost] and 2.05 ug/ml [IM prime(-PEI)/boost]. No specific antibody responses were detected after IN DNA(-PEI) prime/boost vaccination or IN protein vaccination alone indicating an important role for PEI for successful DNA delivery to mucosal surfaces and the inability of antigen alone to prime immune responses. At week 0, none of the treatment groups exceeded the baseline threshold for mucosal antigen-specific IgG or IgA antibody positivity. For mucosal IgG and IgA, the values at week 0 were below 0.274 ng/ml and 0.091 ng/ml respectively. Both the IN prime(+PEI)/boost and the IM prime(-PEI)/boost groups generated antigen-specific mucosal IgG (774.5±884, **p = 0.0079 and 413.5±837 ng/ml, **p = 0.0097 respectively) and IgA (382.2±509 ng/ml, **p = 0.0097 and 20.13±11, **p = 0.0097) in the vaginal vault (**Figs. 1c,d**). Interestingly although both the IM prime(-PEI)/boost and IN prime(+PEI)/boost strategies generated similar antibody concentrations in the systemic compartment, the IN prime(+PEI)/boost regimen generated significantly more specific IgA in the vaginal vault compared to the IM prime(-PEI)/boost group (*p = 0.0317). Collectively this data suggests that a single 20 µg DNA vaccination applied to mucosal surfaces can prime for subsequent protein boost vaccinations but may be relatively inefficient. Therefore we anticipate that multiple DNA primes may be necessary to attain strong humoral responses to a DNA priming candidate.

### Impact of Administration Route on Systemic Humoral Responses induced by Mucosal DNA Prime - Protein Boost Regimens

**Figure 2:**
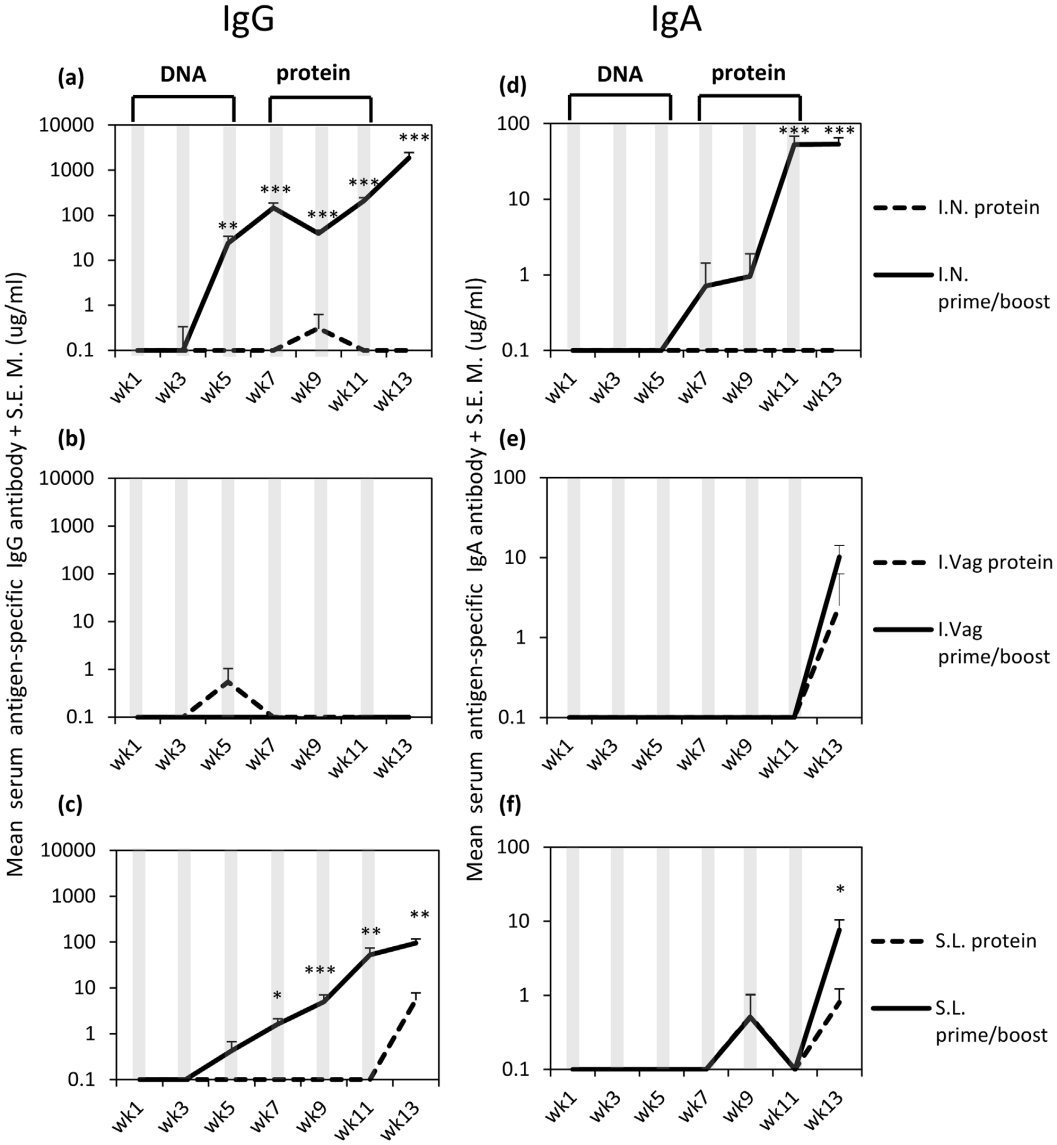
A triple DNA prime, triple protein boost regimen results in antigen-specific IgG and IgA in serum samples. Serum antigen-specific IgG and IgA antibody responses (n = 8 mice per group) were assessed by ELISA one week after each immunisation until study week 13. Mice were immunised every two weeks, with either three 20 µg CN54-gp140 polyplex prime vaccinations in a final 15 µl (IN, I.Vag and SL) volume, followed by three 20 µg gp140 protein boost vaccinations, or with just three 20 µg gp140 protein vaccinations. Immunisations are indicated by vertical shaded lines. Lines represent either protein immunisation (**^…^**) or DNA prime/boost vaccination groups (**−**). Antibody results are expressed as geometric group means (ug/ml+SEM). Statistical significance was assessed using Mann Whitney U test with ***p<0.0005; **p<0.005; *p<0.05. Data are representative of two independent experiments.

Having shown that polyplex inoculation, encoding a model trimeric gp140 transgene product, primed the nasal mucosa for antigen-specific humoral responses, we used PEI for all further mucosal applications of DNA complexes. We sought to determine the impact of the route of administration on mucosal immune priming. Here we delivered polyplexes to the IN, SL or I.Vag mucosal surfaces and evaluated the induced humoral responses. Vaccinations consisted of either three doses of 20 µg CN54-gp140 DNA+PEI followed by three doses of 20 µg recombinant gp140 protein by the same route or recombinant gp140 protein in the absence of any DNA priming vaccinations (**Fig. S2**). Priming of CN54-gp140 specific IgG humoral immune responses was observed in animals that received DNA via the IN and SL routes (**Figs. 2a, c**). After the second DNA immunisation (week 3), IgG responses were elevated above baseline levels in response to the IN and SL regimens respectively. The SL group attained significant levels of IgG during study week 7, one week after the third DNA vaccination and both the IN and SL groups IgG responses continued to rise until the end of the study (week 13). At week 13, peak serum antigen-specific IgG concentrations of 1872 µg/ml (***p = 0.0006) and 95.33 µg/ml (**p = 0.006) were attained after IN and SL prime/boost vaccinations (**Figs. 2a, c**). As before, IN immunisation with protein alone did not generate detectable antigen-specific IgG responses. In addition, priming with 20 µg DNA I.Vag followed by protein boost vaccination or by vaccinating by protein alone did not generate any significant serum antigen-specific IgG responses throughout the study (**Fig. 2b**). All animals in the IN and SL CN54-gp140 DNA prime - recombinant gp140 protein boost groups had a 100% seroconversion rate while only 50% of the mice in the SL protein alone group generated antigen-specific IgG responses.

At week 13, antigen-specific IgA was detected in serum after both IN and SL prime boost vaccination regimens (**Figs. 2d, f**). Elevated levels of serum IgA were detected in some animals after the third DNA was delivered IN, but it was only after the second protein boost that significant levels of IgA were detected (**Figs. 2d**). After I.Vag inoculation, no significant serum IgA responses were detected although some animals did produce low amounts of antigen-specific IgA antibody (**Figs. 2e**). Peak serum antigen-specific IgA concentrations were attained at study week 13 with values of 53.6 µg/ml (***p = 0.0006), 10.19 µg/ml and 7.6 µg/ml (*p = 0.0246) for the IN, I.Vag and SL routes respectively. All animals in the IN group seroconverted while 85% of animals in the SL group and 50% of the I.Vag group generated antigen-specific IgA responses.

### Intranasal DNA Immunisation is Equivalent to or Superior to other Routes of Immunisation for Eliciting Systemic Antibody

**Figure 3:**
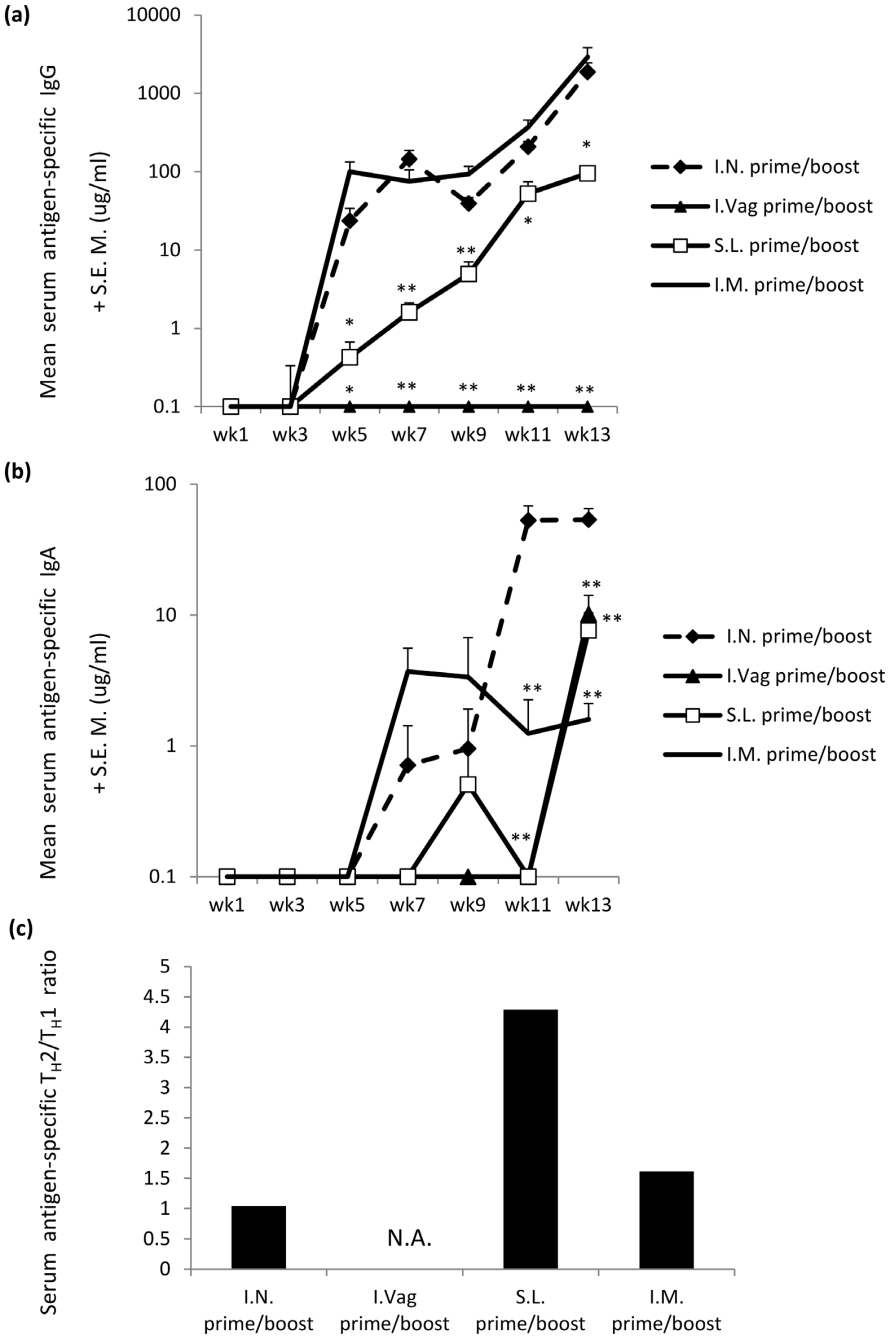
The route of prime/boost vaccination significantly alters serum antigen-specific antibody responses. Serum antigen-specific IgG (**a**) and IgA (**b**) antibody (µg/ml+SEM) responses (n = 8 mice per group) were assessed by ELISA one week after each immunisation until study week 13. The different prime-boost vaccination groups are compared to the IN prime-boost vaccine group for assessment of statistical significance (Wilcoxon non-parametric paired t test). Serum antigen specific IgG1 and IgG2a concentrations (**c**) were assessed during study week 13. The IgG1 and IgG2a ratios were derived by dividing the IgG1 concentration by the IgG2a concentration of each mouse. Antibody results are expressed as geometric group means (µg/ml+data set variance). Statistical significance was assessed using Mann Whitney U test with **p<0.005; *p<0.05 and N.A. = not applicable.

To date, parenteral immunisation, and in particular IM vaccination, is the most common form of vaccine delivery. To evaluate the mucosally delivered vaccine strategies against a standard delivery method we compared the IN DNA prime – protein boost vaccination regimen to the SL and I.Vag routes as well as the more conventional intramuscular (IM) route. The IN and IM regimens, both of which had 100% seroconversion rates by the end of the study, generated virtually identical serum IgG responses, which were found not to be statistically different throughout the study (**Fig. 3a**). However, when comparing the IN vaccination regimen with the SL route, significantly more serum IgG was attained from week 5 to week 13 (*p = 0.0391). This was also the case when comparing the IN and the I.Vag regimens where IN vaccination produced significantly more IgG by week 5 and this was maintained until week 13 (*p = 0.0156). Statistically higher concentrations of specific-IgA were recorded in sera when comparing the IN treatment group and the I.Vag (**p = 0.0078), SL (**p = 0.0078) or IM (**p = 0.0078) groups during study week 11 (**Fig. 3b**).

### The IgG1/IgG2a Antibody Bias is Affected by the Site of Topical Mucosal Application

To gain insight as to the differing effects of mucosally applied DNA prime-protein boost vaccination regimens on the serum antibody response, the IgG1/IgG2a ratios were assessed. As expected, topical application of plasmid DNA followed by recombinant protein boosts to either the IN or SL mucosae generated antigen-specific serum IgG1 and IgG2a immune responses. When analysed as a ratio, the IN inoculation group generated an evenly distributed IgG1/IgG2a response (ratio = 1.042) while the IM vaccination group generated higher concentrations of IgG2a, indicative of a T_H_1 skew (ratio = 1.614). Intriguingly, SL application of the regimen resulted in a clearly biased IgG1 (or T_H_2 skew) antibody response with a ratio of 4.3 (**Fig. 3c**). No IgG responses were detected after I.Vag immunisation therefore no assessment of IgG1 or IgG2a bias was conducted.

### Mucosal DNA Prime - Protein Boost Regimens Elicit Specific Mucosal IgG and IgA

**Figure 4:**
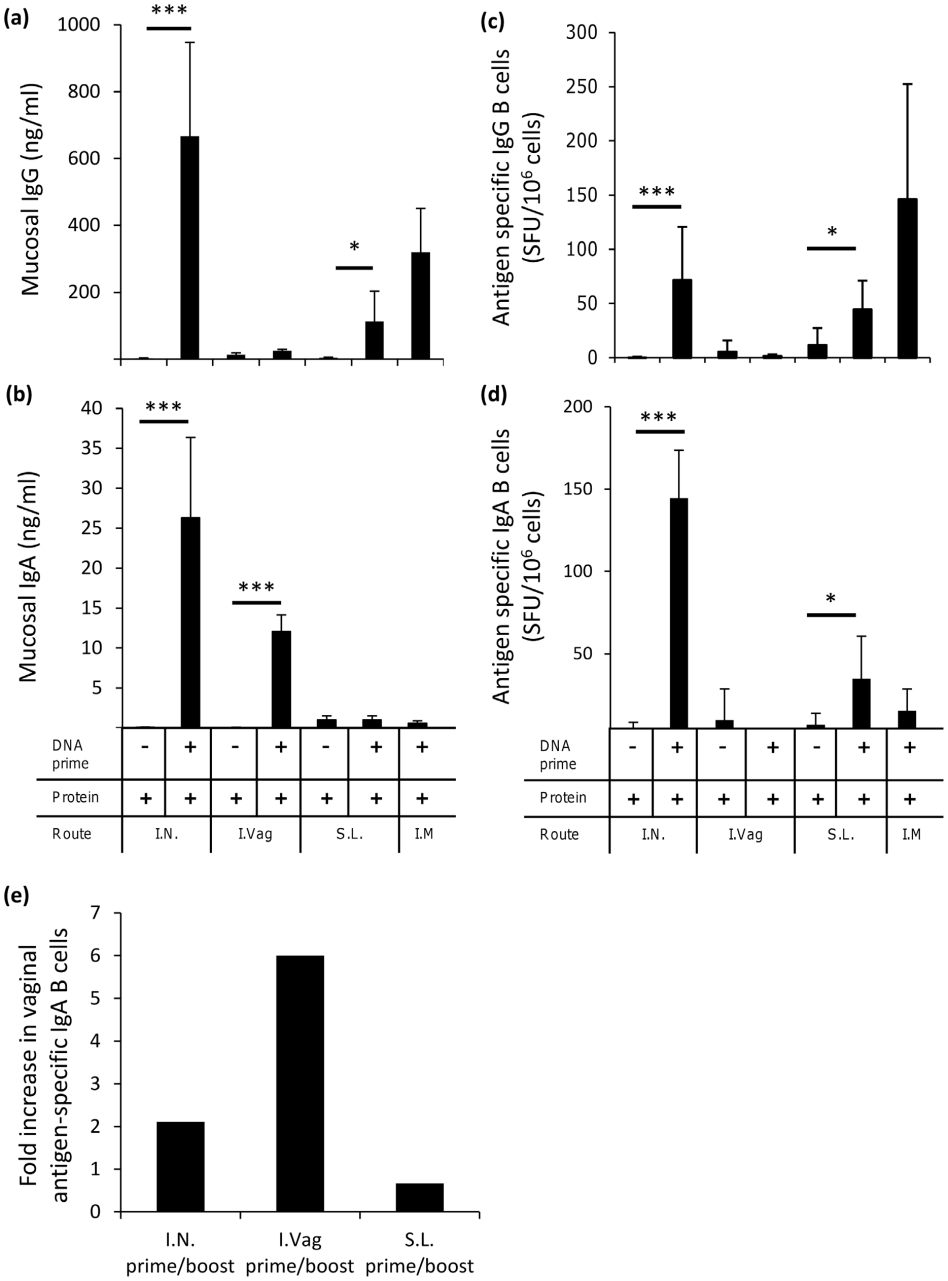
Prime/boost vaccination route impacts vaginal mucosal antibody levels and antigen reactive B cells. Mucosal antigen-specific IgG (**a**) and IgA (**b**) titres from vaginal mucosal lavage were assessed (n = 8 per group) at the end of the study (week 13). Antibody results are expressed as group means (µg/ml+SEM). At the end of the study, antibody secreting IgG (**c**) and IgA (**d**) cells were obtained from excised spleens and plated at a density of 5×10^6^ cells/ml in a commercial IgG or IgA ELISPOT plate. Samples were stimulated with 5 µg/ml gp140 antigen for 16 h and developed as per the manufacturer’s instructions. Results represent group means (n = 8 mice per group) of IgG or IgA Spot Forming Units (SFU)/million antigen stimulated cells (+ SEM). Antigen-specific IgA secreting B cells from enzymatically digested and mechanically disrupted vaginal tissue (**e**) were determined using a direct ELISPOT assay at the end of the study (week 13). Results represent pooled samples and are expressed as fold increase in SFU/million antigen stimulated cells (+ SEM) as compared to negative controls. Statistical significance was assessed using Mann Whitney U test with ***p<0.0005, **p<0.005 and *p<0.05. Data are representative of two independent experiments or are pooled from two experiments.

Protection against mucosally acquired viral infections is likely to require the presence of local antigen-specific antibody to mediate immunological exclusion, viral aggregation or neutralisation. To examine vaccine mediated humoral responses at mucosal surfaces we evaluated vaccine antigen-specific antibody responses following DNA prime-protein boost in vaginal lavage. This allowed for a repeated and non-terminal method to assess mucosal antibody levels throughout the study. Vaccination resulted in antigen-specific mucosal IgG being detected in vaginal lavage following IN (666.4 ng/ml, ***p = 0.0002), and SL (112 ng/ml, *p = 0.049) prime-boost applications while I.Vag vaccination did not generate any significant levels of IgG (**Fig. 4a**). Here 100% and 87.5% seroconversion rates were achieved for the IN and SL prime boost vaccine groups. An IM prime-boost vaccination regime also elicited antigen-specific IgG in vaginal lavage with 100% of the vaccine recipients seroconverting (319.8 ng/ml, *p = 0.0104). Immunisation via the IN route elicited the greatest IgA response (26.3 ng/ml, ***p = 0.0002) with a 100% seroconverion rate while the I.Vag route also generated significant IgA responses (12.04 ng/ml, ***p = 0.0002) and a 62.5% seroconversion rate. Both SL and IM immunisation were ineffective at producing antigen-specific IgA in mucosal vaginal lavage with 50% and 37.5% seroconverting respectively (**Fig. 4b**).

### A DNA Prime - Protein Boost Regimen Generates Splenic B Cell Responses via the Intranasal and Sublingual but not the Intra-vaginal Route

We next examined the CN54-gp140 antigen-specific splenocyte B cell responses using an antigen-specific ELISPOT. Vaccination via the IN and SL routes generated significant numbers of IgG^+^ CN54-gp140 antigen-specific B cells, measured as spot forming units (SFU) in the ELISPOT assay, relative to their unprimed protein controls (71.8 SFU/million cells, ***p = 0.0002 and 44.57 SFU/million cells, *p = 0.0175 respectively) while no responses were recorded for the DNA primed or unprimed I.Vag inoculated animals (**Fig. 4c**). A similar response pattern for antigen-specific IgA^+^ SFU was observed with both the IN (144.5 SFU/million cells, ***p = 0.0002) and SL (34.9 SFU/million cells, *p = 0.0379) regimens (**Fig. 4d**). Within this study, we did not detect any systemic antigen-specific B cells in those animals that had received the I.Vag inoculations, despite the presence of statistically significant levels of CN54-gp140 specific antibody within the vaginal mucosal secretions of this group (**Fig. 4d**). A low number of CN54-gp140 specific IgA^+^ SFU were recorded for the IM control route group (15.4 SFU/million cells), while the same group generated 146.3 SFU/million antigen-specific IgG^+^ cells (**Figs. 4c,d**).

Antigen-specific IgA^+^ SFU were also enumerated from pooled single cell suspensions (n = 5) directly isolated from vaginal mucosal tissue. After IN and I.Vag DNA prime-boost immunisation, a 2- and 6-fold increase respectively in the IgA^+^ CN54gp140-specific B cell numbers were recorded when comparing them to non-DNA primed animals. The lack of antigen-specific B cells in the periphery but their presence within the vaginal tissue strongly suggests local mucosal antibody production. This indicates that both local priming and immunological linkage can be employed to increase antigen-specific IgA responses within the vaginal mucosae (**Fig. 4e**). Sublingual vaccination did not increase the numbers of antigen-specific B cells within vaginal preparations.

### Intranasal and Sublingual DNA Prime - Protein Boost Regimens Provide Protection Against Influenza Challenge

**Figure 5:**
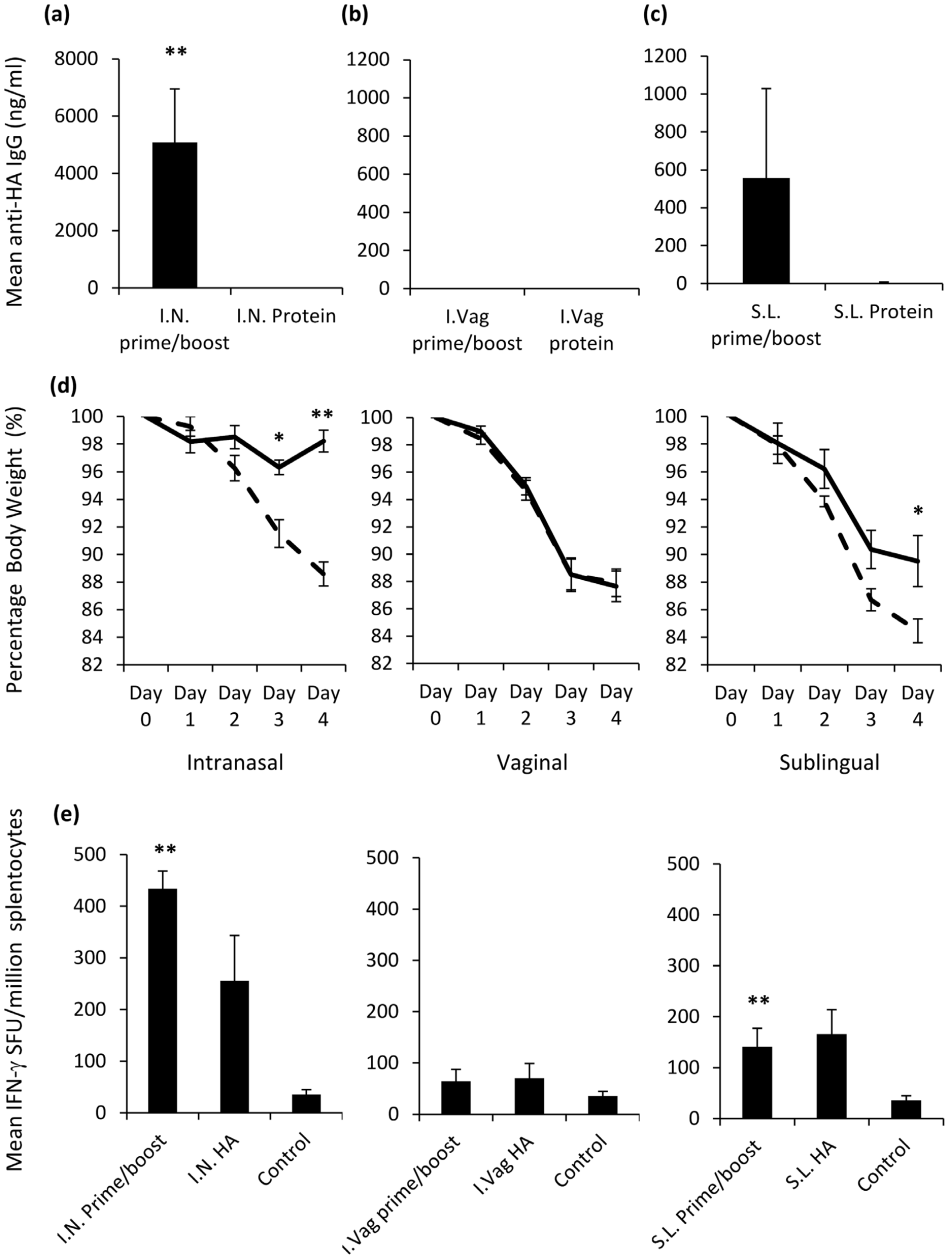
Nasal and sublingual vaccination results in protection against Influenza challenge infection. Serum-antigen-specific IgG antibody responses (n = 6 per group) were recorded one week after 3 DNA primes and a single HA boost (**a**, **b** and **c**). Specifically, a 20 µg/30 µl volume of plasmid DNA was administered three times, every two weeks, before a single 10 µg/30 µl HA protein boost. Serum anti-HA antibody concentrations are expressed as group means (ng/ml+SEM). One week after the final vaccination, mice were infected with 5HA units of Influenza X-31 in 100 µl (**d**). Body weights for DNA prime protein boost vaccinations (−) and single protein vaccination (−) groups are expressed as a mean percentage of total weight relative to day 0. Mean IFN-γ T cell responses were assessed on day 4 post infection using a commercial ELISPOT assay coated with HA protein (**e**). Results represent the means of (n = 6) IFN-γ SFU/million splenocytes on day 4 post-infection. Statistical significance was assessed using Mann Whitney U test with **p<0.005 and *p<0.05. Data are representative of two independent experiments.

In the absence of a small animal model for HIV-1 infection, we tested the protective efficacy of the vaccination regimens delivered via different mucosal routes using a mucosally acquired influenza infection-challenge model. Here the challenge was provided by the murine adapted reassortment X-31 (H3N2) influenza strain. As the delivery of multiple doses of this influenza HA protein via the intranasal route can provide protection against infection (data not shown), we altered the vaccination regimen to incorporate only a single protein boost following a triple DNA prime (**Fig. S3**). In this way, the effective priming of mucosal surfaces through the application of DNA polyplexes could be evaluated. The application of DNA resulted in detectable levels of antigen-specific IgG in serum prior to IN protein boost vaccination (100% seroconversion); and significantly elevated levels [5088 ng/ml (**p = 0.0022)] of specific IgG being detected after IN protein boost **(** **Fig. 5a** **)**. Although, elevated levels of serum IgG could be detected after SL protein boost vaccination **(** **Fig. 5c** **)**, this was found not to be significant (66% seroconversion). I.Vag protein boost vaccination did not lead to any detectable antigen-specific IgG in serum **(** **Fig. 5b** **)**. Interestingly, viral challenge resulted in protection in the IN (**p = 0.0022) and SL (*p = 0.026) vaccination regimens on day 4 post infection respectively. No protection was seen after I.Vag vaccination **(** **Fig. 5d** **)**. As the mucosal prime-boost vaccination strategy was designed to prevent infection, no further monitoring of weight loss was assessed past day 4; a time point previously established to distinguish statistical significance between protected and unprotected controls. Moreover, both the IN (433.3 SFU/million cells, **p = 0.0043) and SL (141.3 SFU/million cells, **p = 0.0087) DNA prime boost vaccination regimen resulted in significantly elevated IFN-γ T cell responses compared to the unimmunised controls **(** **Fig. 5e** **)**.

## Discussion

It is anticipated that mucosally applied vaccination strategies will be superior to conventional parenteral based vaccination modalities for providing protective local mucosal responses against invading pathogens [18]. Furthermore, the concept of mucosal linkage allows for discrete but linked mucosal routes of immunisation to elicit protective local immune responses through the selective recruitment and homing of activated immune cells [19], [20]. Previous studies utilising HIV-1 gp140 antigens have demonstrated their limited immunogenicity upon injection [21], while we and others have shown unadjuvanted trimeric HIV-1 CN54gp140 antigen to be ineffective at generating antigen-specific responses when applied topically to the vagina and nasal mucosa [22], [23]. Therefore in the current study we explored the potential of mucosal DNA vaccination to improve the local immune priming of vaccine antigen-specific responses. Moreover, we compared and contrasted the humoral immune responses elicited following DNA prime and protein boost immunisation regimens at different target mucosal surfaces. While a number of DNA prime protein boost vaccination strategies have been published using a range of HIV-1 antigen constructs, the majority involve the parenteral delivery of the DNA prime or viral vector component [24]–[27]. To our knowledge, few studies have directly compared the intranasal, vaginal and sublingual routes for their abilities to be immunologically primed by a gp140 expressing plasmid. This makes associations with other studies difficult. Certainly, of the few studies employing the mucosal route of delivery for both the prime and boost components, there is agreement that the plasmid DNA facilitates augmented immune responses. For instance, the use of DNA-VLP prime boost immunisation has been shown to significantly increase serum anti-env antibody titres, T cell proliferation and IFN-γ production when compared to VLP-VLP prime boost vaccine regimens [28].

In this study we found that the DNA prime protein boost vaccination regimen applied to the nasal mucosa elicited both antigen-specific IgG and IgA and antigen-specific B cells in systemic and mucosal compartments. This contrasted to the vagina as a site of inoculation, which only generated serological IgA, mucosal IgA and a 6-fold increase in the numbers of antigen-specific IgA producing B cells in the vagina but none in the spleen. Finally, we found that sublingual delivery resulted in mucosal IgG and low level IgG and IgA B cell responses in spleen along with a biased IgG2a profile but no increase in antigen-specific vaginal B cell numbers. Collectively these results allow us to conclude that nasal immunisation is superior to the other forms of mucosal delivery tested; for generating humoral responses in the mouse model. However, if local responses in the vagina are required, then these studies show that vaginal delivery can cause greater local recruitment of antigen-specific B cells compared to IN and SL regimens.

In the mouse, the ability of the vaginal epithelium to participate in the luminal transcytosis of IgA has been shown to be dependent on the stage of the estrus cycle. Furthermore the expression of pIgR was previously found to be increased in Depo-treated mice [29]. Thus the observed difference between the IN and I.Vag mucosal IgA concentrations after DNA prime-boost vaccination may be due systemic dissemination. The observed increase in lymphocyte recruitment to the vagina is supported by previous observations by Marks *et al*., 2011 [30]. Here they used adoptively transferred OVA-specific TCR transgenic T cells and showed that I.Vag immunisation using OVA conjugated to the potent CT adjuvant generated T cell recruitment to the vagina and enhanced CD4^+^ T cell responses in the draining lymph nodes. In our study, no T cell responses were assayed and the measured breadth of the B cell humoral response after topical vaginal vaccination is restricted to antigen-specific IgA and does not encompass IgG. In addition, IgA was seen only after multiple protein boosts.

Using an influenza challenge system, we were able to assess the effective functionality of plasmid DNA mediated priming through mucosal surfaces. As expected, the data shows that effective immune priming could be achieved after IN DNA vaccination, resulting in detectable antibody levels upon adjuvant-free protein boosting. Moreover, this data suggests that the SL route can also be utilised as a delivery site for plasmid DNA prime vaccination. Previous studies by Song *et al*., 2008 has shown that application of inactivated influenza or live A/PR/8 virus to the SL space can convey protection against challenge infection [31]. Critically they show that administration of substances via the SL route does not lead to trafficking of vaccine components to the CNS, making this route of administration appear safer when compared to IN. While we observed elevated antibody levels in serum after SL vaccination with DNA polyplexes and protein boost, this was not statistically significant. However, as multiple protein SL immunisations in the absence of DNA priming is ineffective against viral challenge (data not shown), it is highly likely that additional protein boosts to the DNA priming protocol would have improved upon this. Critically, after SL vaccination, there was an observed partial protection against influenza challenge in these animals with a statistically relevant reduction in infection-associated weight loss. This has important implications for the vaccine field as this data suggests that, not only is it possible to prime immune responses via the intranasal route but, it may also be possible to successfully deliver DNA and prime immune responses via the sublingual route. While low volume vaccine formulations are preferable to prime specific tissues and avoid formulation dissemination, it should be mentioned that within this study we used a 30 µl vaccine formulation as opposed to the initial 15 µl volumes. This was done to compensate for DNA-PEI complex precipitation. Therefore, as volumes greater than 20 µl have the opportunity to 1) prime the lower respiratory tract as well as the NALT after IN administration, 2) increase the chance of swallowing after SL administration and 3) increase the chance of leakage after vaginal administration, this should be taken into account.

Taken together these findings may have important implications for vaccine design where protection of at-risk mucosal tissues is desired. While these current studies have been conducted in the murine model, future studies will need to be carried out to evaluate the different routes of mucosal vaccination in the context of DNA prime, recombinant protein boost vaccine strategies in humans. Furthermore, it would be interesting to see how concomitant delivery of prime-boost vaccination strategies to multiple different mucosal tissues might influence immune responses. This could have a significant impact for prophylactic disease prevention of at risk tissues, such as the mucosa; where heightened local mucosal B cell and antibody responses may be beneficial.

## Supporting Information

Figure S1 **Vaccination schedule for study 1.** Intranasal DNA prime – protein boost vaccination studies were carried out on female BALB/c mice. Mice (n = 6 per group) were immunised at two week intervals (red arrows) with a single gp140 DNA prime vaccination (20 µg) followed by triple gp140 recombinant protein boost (20 µg) vaccinations. Blood sampling for antigen-specific antibody determination was carried out as indicated.
(TIF)

Figure S2 **Vaccination schedule for study 2.** Intranasal, sublingual and intravaginal immunisation using DNA prime – protein boost vaccination regimens were carried out on medroxyprogesterone treated female BALB/c mice. Mice (n = 6 per group) were immunised at two week intervals (red arrows) with a triple DNA prime vaccination (20 µg) regimen followed by triple recombinant gp140 protein boost (20 µg) vaccinations. Blood sampling for antigen-specific antibody determination was carried out as indicated.
(TIF)

Figure S3 **Vaccination schedule for Influenza challenge study 3.** Intranasal DNA prime – protein boost vaccination studies were carried out on female BALB/c mice. Mice (n = 6 per group) were immunised at two week intervals (red arrows) with a triple DNA prime vaccination (20 µg) followed by a single recombinant HA protein boost (10 µg) vaccination. Blood sampling for antigen-specific antibody determination was carried out as indicated.
(TIF)
