# Supplementary figures and images for: Mucosal Application of gp140 Encoding DNA Polyplexes to Different Tissues Results in Altered Immunological Outcomes in Mice
Source: PLoS One. 2013 Jun 24;8(6):e67412. doi: 10.1371/journal.pone.0067412 (PMC3691144; doi:10.1371/journal.pone.0067412)

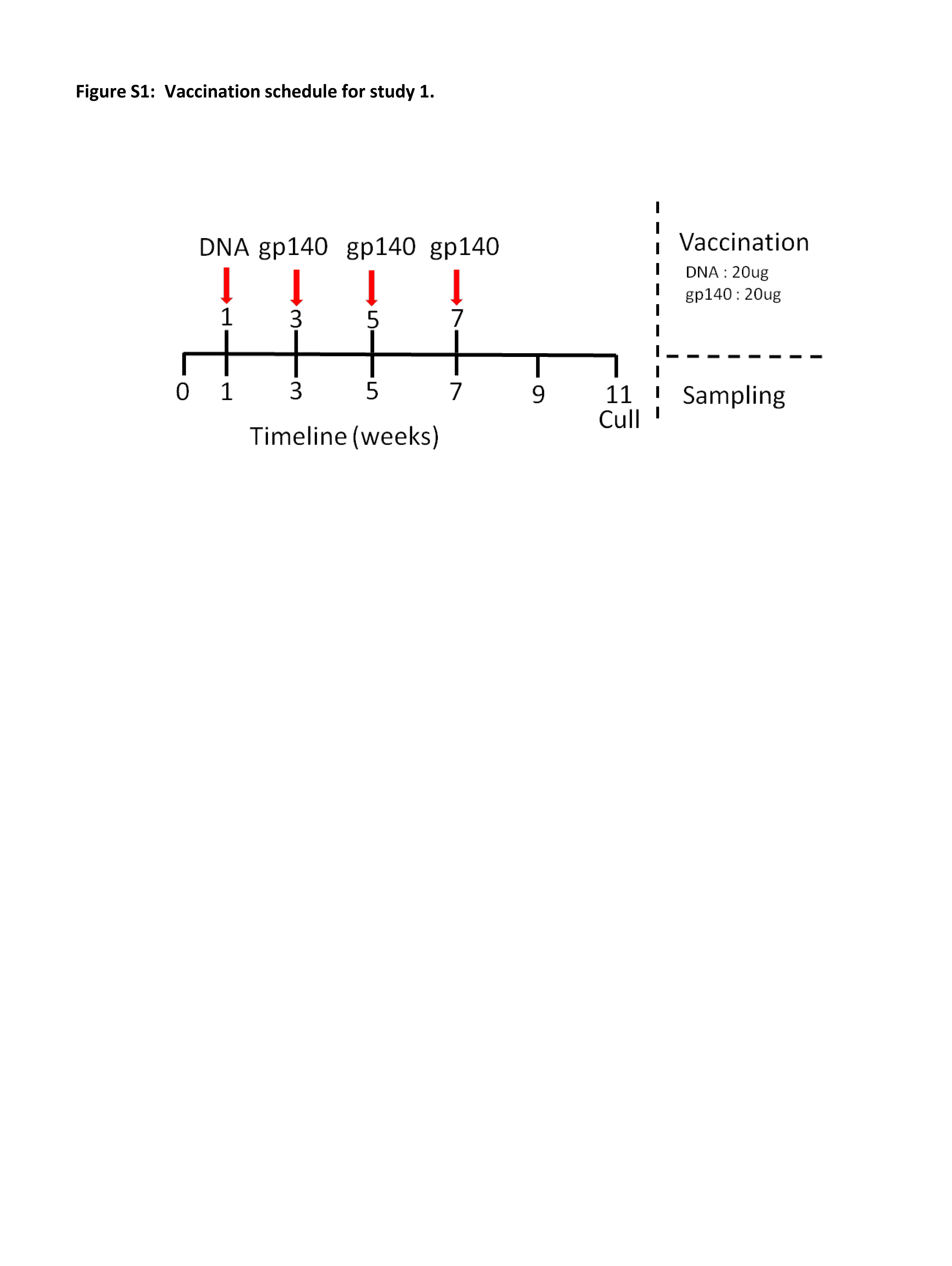

Supplement: Figure S1 — Vaccination schedule for study 1. Intranasal DNA prime – protein boost vaccination studies were carried out on female BALB/c mice. Mice (n = 6 per group) were immunised at two week intervals (red arrows) with a single gp140 DNA prime vaccination (20 µg) followed by triple gp140 recombinant protein boost (20 µg) vaccinations. Blood sampling for antigen-specific antibody determination was carried out as indicated. (TIF) [file pone.0067412.s001.tif]

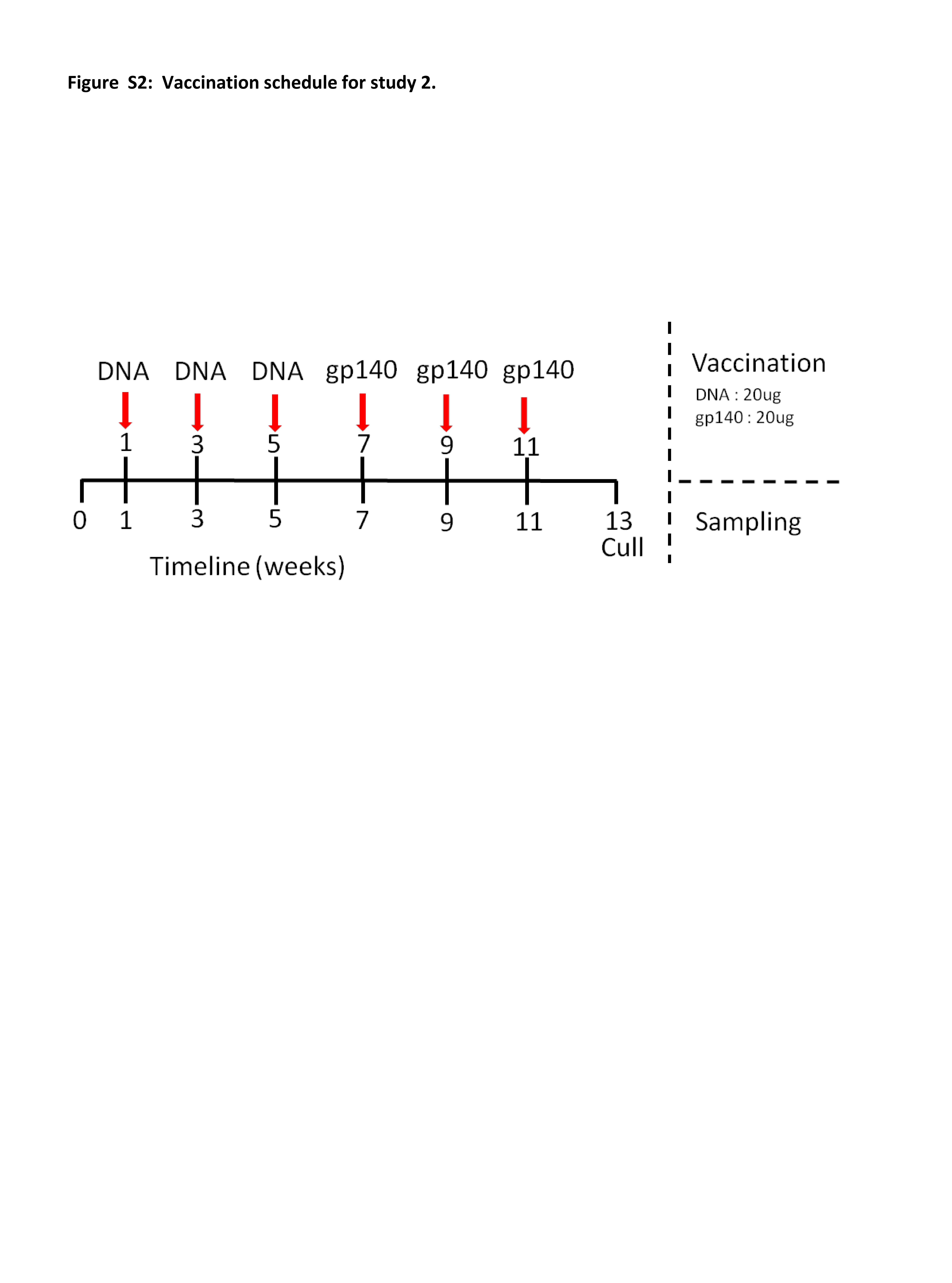

Supplement: Figure S2 — Vaccination schedule for study 2. Intranasal, sublingual and intravaginal immunisation using DNA prime – protein boost vaccination regimens were carried out on medroxyprogesterone treated female BALB/c mice. Mice (n = 6 per group) were immunised at two week intervals (red arrows) with a triple DNA prime vaccination (20 µg) regimen followed by triple recombinant gp140 protein boost (20 µg) vaccinations. Blood sampling for antigen-specific antibody determination was carried out as indicated. (TIF) [file pone.0067412.s002.tif]

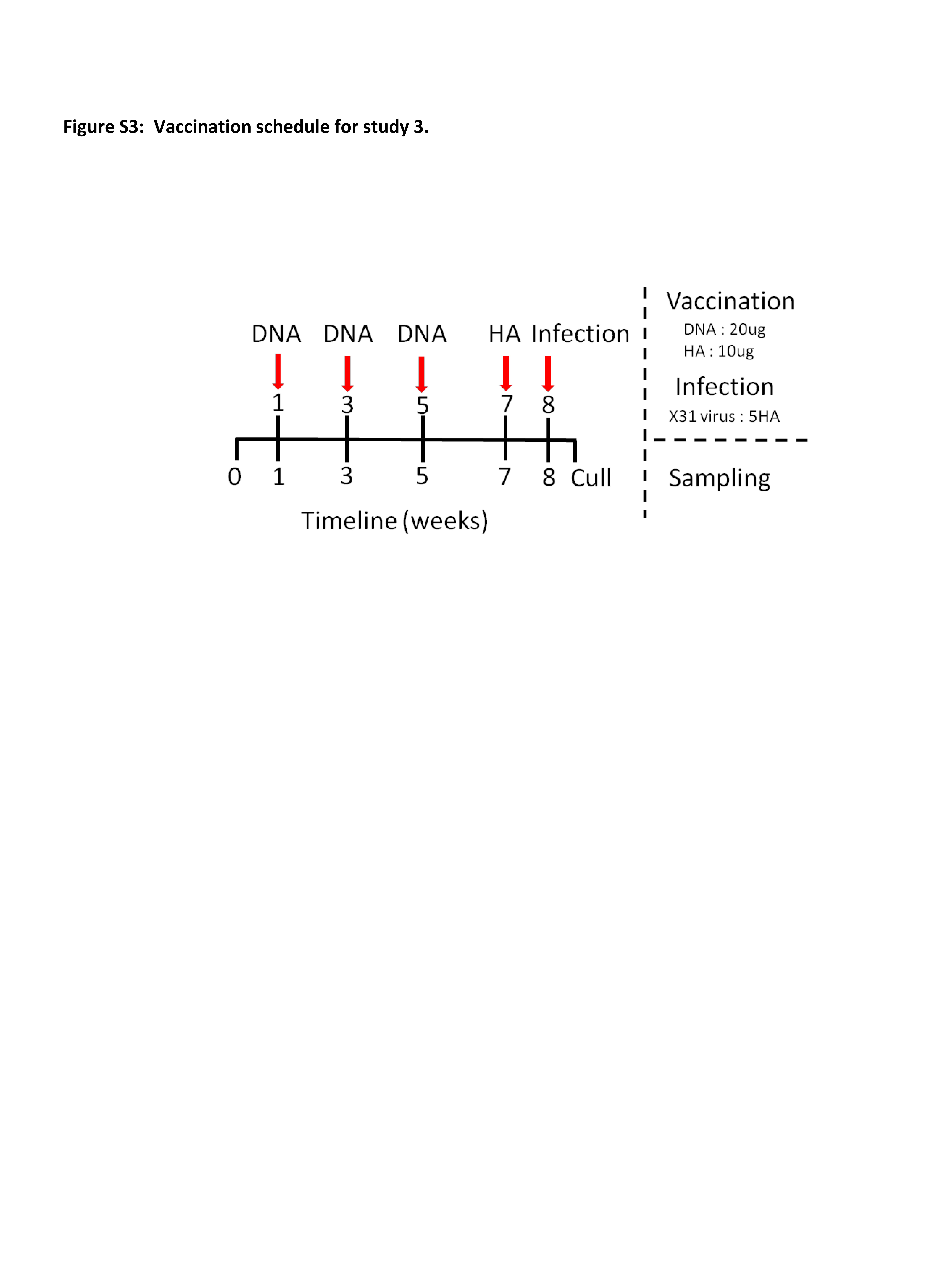

Supplement: Figure S3 — Vaccination schedule for Influenza challenge study 3. Intranasal DNA prime – protein boost vaccination studies were carried out on female BALB/c mice. Mice (n = 6 per group) were immunised at two week intervals (red arrows) with a triple DNA prime vaccination (20 µg) followed by a single recombinant HA protein boost (10 µg) vaccination. Blood sampling for antigen-specific antibody determination was carried out as indicated. (TIF) [file pone.0067412.s003.tif]
